# Professional Jealousy

**Published:** 1892-06

**Authors:** 


### Editorial.

### PROFESSIONAL JEALOUSY.

It is probably true that the human mind never will be free from petty envious feelings, yet it is equally certain that of all classes of the world's workers, professional men should rise superior to their disquieting influence.

If a profession is anything in its tendencies, it is humanitarian. If it fails in this it is dwarfed into a sordid effort for gain. The grosser elements of the human constitution, the legacy, it may be, of the savagism of the past, lead all in degree to entertain the selfish side and demand the largest portion of the loaf, whether that be in the form of knowledge or material benefit.

The fact that there are limitations to all effort seems to be lost sight of in the whirl of active life. Contentment with place is not generally one of the philosophies of mankind; the work of superior intellect is often marred by the unceasing sneers, the sharp retort, the coldness of mediocrity, the man of one talent aspiring to the place of the man with five, and the man with five ambitious for the place of the ten, and each tenacious of their prerogatives and jealous of their real or supposed rights.

It is in the memory of many now living, that there was a time in the history of dentistry when each practitioner was wholly isolated. To open an office for the admission of a fellow-dentist was an unheard-of condescension. To explain any process which might prove of value to a competing operator was an unthought-of extension of charity. Time made its changes in this, but how slowly it came, and through what struggles before visible progress was apparent. How many years before offices were freely opened, and how many more before men would present themselves at gatherings of their fraternity and tell all they knew? Yet the time came, and from that period dentistry rose from its poor condition to one worthy of the respect of all classes. Societies have sprung into being everywhere. The man of isolation is no longer permitted. Hermits in professional life have ceased to be respected. The man who has something to say or something to show must say it or exhibit it. Each must take his part, for it is and must continue to be a busy hive without drones.

Having reached a stage of partial exemption from the evil, it is difficult for the practitioner of this country to comprehend that the old feeling still remains elsewhere, and is as much a source of bitterness in other lands as it was here during the first half of the present century.

It is the tendency of civilization to have periods of retrogression, —a time when, as it were, the one foot forward means the one step backward. It is to be feared that the attempt to build a professional wall around States, by laws which prevent the dentist recognized in one from settling in another, is in this direction, and in line with that which influenced the dentist who refused to permit a brother operator to cross his threshold.

It is not surprising that older civilizations have adopted this system of exclusion, for the avenues of labor are sharply defined in European communities, and it is often a matter of self-preservation to place a barrier to all outside intrusions.

A broader liberality exists with the dentists of this country, in spite of what we regard as but a temporary relapse. They appreciate the good in their own land, and are not ill-disposed to share it with less fortunate neighbors. No one here has reason to envy the older and better-grounded civilization of Great Britain, or the almost matchless scholarship of Germany. No one would debate the question with our English brethren as to the value of a solid collegiate training as a basis of professional study. No one can justly quarrel with the curricula of the dental schools or the higher standard of entrance examinations. A recognized dentist of England or Germany would not here be subjected to the surveillance of the police, at the request of fellow-practitioners, as has been done in the latter country. On the contrary, he would be received politely, and while he might be requested to comply with the law and pass the learned examination of some State Board, he would be accepted as one worthy high consideration.

Our thoughts have tended in this direction ever since the unseemly wrangle of words fully quoted from *The Journal of the British Dental Association* in our last number. The remarkable statements in regard to colleges in the United States, and the perversion of facts in relation thereto, forces a return to the subject and to the spirit which evidently animated the writers.

The gravamen of this whole matter does not seem to have been to set us right in science and collegiate training, but rather to afford a basis for venting a spirit of jealousy of work other than their own. It is the same spirit that made the long contest between the Odontological Society of London and the College of Dentists of England one to be remembered. Jealousy and the spirit of caste served to place a barrier for years to the progress of the dental profession in that country.

The record made by the dentists of this land is one that requires no apology. Dentistry came here from the Mother-Country. It had but few caretakers in that early period, but it grew in strength, and in time became a profession. It does not require a boastful spirit of expression to say that the world is indebted to the United States for dentistry as it stands to-day in civilization. About everything of practical value in appliances and treatment have originated here. Nor has this country been behind in the scientific work so much derided. It is unnecessary to give examples, but we are constrained to cite the fact that it was an American who solved the problem of dental caries, notwithstanding that the scientific minds of both England and Germany had been eagerly endeavoring to interpret the pathological phenomena connected with it for more than a century.

It is but just that these facts should be conceded, and that without reservation or fear of rivalry. The work of this land in this direction has been given freely, and as freely appropriated. It has been the aim to give credit to workers elsewhere, and we need only recall the Herbst testimonials in proof of it, yet it would show a want of the true scientific spirit, if the acknowledgment were not made that in some directions the practitioners of the Old World lead those of this country.

The environments of life on this continent make progress in certain directions more difficult for the professions than in older civilizations. The higher standard, easy of attainment in England, must come slowly here; but it will come, and the superstructure will be built on a sure foundation of practical success.

The lines of separation which divide nationalities must be broken, and in the coming era those will be accorded recognition who can demonstrate that they have worked not for a narrow circle, but for the higher elevation of all races and of all conditions.

A profession should know no bounds to fraternal progress, nor should the intellectual forces common to all nationalities be dwarfed by local prejudices or the conventionalities of race.

